# Bis{4-[(4*H*-1,2,4-triazol-4-yl)iminomethyl]pyridinium} diaquapentanitratocerate(III)

**DOI:** 10.1107/S1600536808043109

**Published:** 2008-12-24

**Authors:** Qiaozhen Sun, Feng Zheng, Xiaodan Sun, Wei Wang

**Affiliations:** a Department of Materials Chemistry, School of Materials Science and Engineering, Central South University, Changsha, 410083, People’s Republic of China

## Abstract

The asymmetric unit of the title compound, (C_8_H_8_N_5_)_2_[Ce(NO_3_)_5_(H_2_O)_2_], contains one independent protonated 4-pyridyl­methyl­eneamino-1,2,4-triazole cation and half of a centrosymmetric [Ce(NO_3_)_5_(H_2_O)_2_]^2−^ anion. The Ce atom coordinated by five NO_3_^−^ anions and two water mol­ecules, exhibiting a distorted 10-coordination. In the crystal structure, inter­molecular O—H⋯N and N—H⋯O hydrogen bonds result in the formation of a supramolecular structure.

## Related literature

For related compounds based on 4-amido-1,2,4-triazoles Schiff base ligands, see: Drabent *et al.* (2003 ▶, 2004 ▶); Wang *et al.* (2006 ▶). For the preparation of the ligand, see: Colautti *et al.* (1971 ▶).
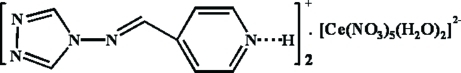


## Experimental

### 

#### Crystal data

(C_8_H_8_N_5_)_2_[Ce(NO_3_)_5_(H_2_O)_2_]
*M* *_r_* = 834.59
Monoclinic, 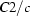

*a* = 10.322 (4) Å
*b* = 16.126 (6) Å
*c* = 17.595 (7) Å
β = 100.107 (4)°
*V* = 2883.2 (19) Å^3^
*Z* = 4
Mo *K*α radiation
μ = 1.69 mm^−1^
*T* = 293 (2) K
0.20 × 0.18 × 0.15 mm

#### Data collection

Bruker SMART CCD area-detector diffractometer
Absorption correction: multi-scan (*SADABS*; Bruker, 2000 ▶) *T*_min_ = 0.682, *T*_max_ = 0.781
5464 measured reflections
2374 independent reflections
2164 reflections with *I* > 2σ(*I*)
*R*_int_ = 0.039

#### Refinement

*R*[*F* ^2^ > 2σ(*F* ^2^)] = 0.024
*wR*(*F* ^2^) = 0.051
*S* = 1.02
2374 reflections
231 parameters
3 restraints
H atoms treated by a mixture of independent and constrained refinement
Δρ_max_ = 0.47 e Å^−3^
Δρ_min_ = −0.39 e Å^−3^

### 

Data collection: *SMART* (Bruker, 2000 ▶); cell refinement: *SAINT* (Bruker, 2000 ▶); data reduction: *SAINT*; program(s) used to solve structure: *SHELXTL* (Sheldrick, 2008 ▶); program(s) used to refine structure: *SHELXTL*; molecular graphics: *SHELXTL*; software used to prepare material for publication: *SHELXTL*.

## Supplementary Material

Crystal structure: contains datablocks I. DOI: 10.1107/S1600536808043109/wk2094sup1.cif

Structure factors: contains datablocks I. DOI: 10.1107/S1600536808043109/wk2094Isup2.hkl

Additional supplementary materials:  crystallographic information; 3D view; checkCIF report

## Figures and Tables

**Table 1:** Hydrogen-bond geometry (Å, °)

*D*—H⋯*A*	*D*—H	H⋯*A*	*D*⋯*A*	*D*—H⋯*A*
O*W*1—H*W*1*A*⋯N7	0.85 (2)	1.96 (2)	2.805 (3)	176 (3)
O*W*1—H*W*1*B*⋯N8^i^	0.85 (2)	2.03 (3)	2.876 (3)	178 (3)
N4—H4*B*⋯O4^ii^	0.86	1.95	2.804 (3)	172
N4—H4*B*⋯N2^ii^	0.86	2.69	3.518 (4)	162

Symmetry codes: (i) 
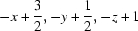
; (ii) 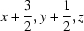.

**Fig. 1.**
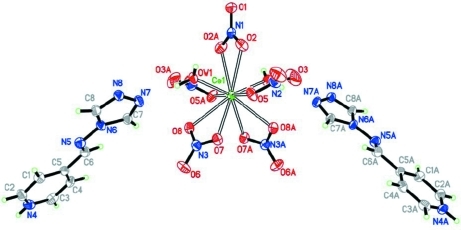
The molecular structure of the title molecule, with the atom-numbering scheme. Displacement ellipsoids are drawn at the 30% probability level. [symmetry codes: (A) 1 - x, y, 0.5 - z.]

**Fig. 2.**
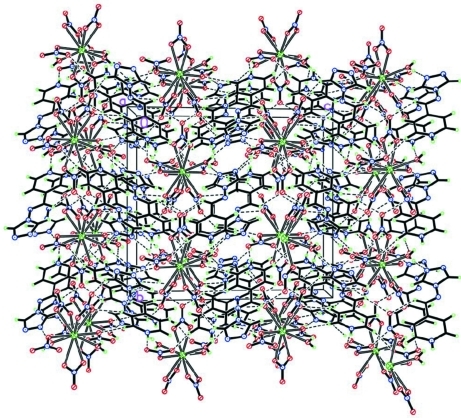
A packing diagram of the title compound along a axis. The dash lines indicate hydrogen bonding.

## supplementary crystallographic information

### Comment

In recent decades, metal coordination polymers containing 1,2,4-triazoles and their derivatives have been studied widely due to their versatile bridging modes with metal ions. Relatively few  structurally characterized compounds based on 4-amido-1,2,4-triazoles Schiff base ligands have been reported (Drabent *et al.*, 2004 and 2003; Wang *et al.*, 2006) and these compounds exhibit  dinuclear and tetranuclear structures. In this work,  4-Pyridylmethyleneamino-1,2,4-triazole coordinated with metal cerium is shown to generate a new coordination compound and its crystal structure reported.

As depicted in Fig.1, the Ce ion in this complex is ligated by eight oxygen atoms from five NO_3_^-^ anions and two from H_2_O molecules. And three NO_3_^-^ anions are bound to Ce ion in bidentate fashion, while the other two in monodentate one. The nitrogen atoms of Schiff base ligand are not involved in the coordination to the Ce center as we supposed. The [Ce(NO_3_)_5_(H_2_O)]^2-^ unit and the ligands are linked through the O—H···N and N—H···O hydrogen bonds, forming a three dimensional network.

### Experimental

The ligand was prepared according to the reported literature (Colautti *et al.*, 1971). The ligand (0.1 mmol, 0.017 g) and (NH_4_)_2_Ce(NO_3_)_6_ (0.1 mmol, 0.055 g) were mixed in acetonitrile and methanol of 1:1. After stirring at room temperature for four hours, the yellow solution was filtered and evaporated at room temperature. A few days later the red block crystals were obtained.

### Refinement

All of the non-hydrogen atoms were refined with anisotropic thermal displacement parameters. The hydrogen atoms on the water coordinated to the Ce atom were located in the difference Fourier, restraints applied to distance and angles and then refined. The positions of other hydrogen atoms were fixed geometrically at calculated distances and allowed to ride on the parent non-hydrogen atoms.

### Crystal data

**Table:**

(C_8_H_8_N_5_)_2_[Ce(NO_3_)_5_(H_2_O)_2_]	*F*(000) = 1660
*M**_r_* = 834.59	*D*_x_ = 1.923 Mg m^−^^3^
Monoclinic, *C*2/*c*	Mo *K*α radiation, λ = 0.71073 Å
*a* = 10.322 (4) Å	Cell parameters from 4413 reflections
*b* = 16.126 (6) Å	θ = 2.4–27.9°
*c* = 17.595 (7) Å	µ = 1.69 mm^−^^1^
β = 100.107 (4)°	*T* = 293 K
*V* = 2883.2 (19) Å^3^	Block, red
*Z* = 4	0.2 × 0.18 × 0.15 mm

### Data collection

**Table:**

Bruker SMART CCD area-detector diffractometer	2374 independent reflections
Radiation source: fine-focus sealed tube	2164 reflections with *I* > 2σ(*I*)
graphite	*R*_int_ = 0.039
φ & ω scans	θ_max_ = 25.0°, θ_min_ = 2.4°
Absorption correction: multi-scan (*SADABS*; Bruker, 2000)	*h* = −8→12
*T*_min_ = 0.682, *T*_max_ = 0.781	*k* = −19→17
5464 measured reflections	*l* = −20→20

### Refinement

**Table:**

Refinement on *F*^2^	Primary atom site location: structure-invariant direct methods
Least-squares matrix: full	Secondary atom site location: difference Fourier map
*R*[*F*^2^ > 2σ(*F*^2^)] = 0.024	Hydrogen site location: geom, H2O from difmap
*wR*(*F*^2^) = 0.051	H atoms treated by a mixture of independent and constrained refinement
*S* = 1.02	*w* = 1/[σ^2^(*F*_o_^2^) + (0.0252*P*)^2^] where *P* = (*F*_o_^2^ + 2*F*_c_^2^)/3
2374 reflections	(Δ/σ)_max_ = 0.001
231 parameters	Δρ_max_ = 0.47 e Å^−^^3^
3 restraints	Δρ_min_ = −0.39 e Å^−^^3^

### Special details

**Table:**

Geometry. All e.s.d.'s (except the e.s.d. in the dihedral angle between two l.s. planes) are estimated using the full covariance matrix. The cell e.s.d.'s are taken into account individually in the estimation of e.s.d.'s in distances, angles and torsion angles; correlations between e.s.d.'s in cell parameters are only used when they are defined by crystal symmetry. An approximate (isotropic) treatment of cell e.s.d.'s is used for estimating e.s.d.'s involving l.s. planes.
Refinement. Refinement of *F*^2^ against ALL reflections. The weighted *R*-factor *wR* and goodness of fit *S* are based on *F*^2^, conventional *R*-factors *R* are based on *F*, with *F* set to zero for negative *F*^2^. The threshold expression of *F*^2^ > σ(*F*^2^) is used only for calculating *R*-factors(gt) *etc*. and is not relevant to the choice of reflections for refinement. *R*-factors based on *F*^2^ are statistically about twice as large as those based on *F*, and *R*- factors based on ALL data will be even larger.

### Fractional atomic coordinates and isotropic or equivalent isotropic displacement parameters (Å^2^)

**Table:**

	*x*	*y*	*z*	*U*_iso_*/*U*_eq_	
Ce1	0.5000	0.326614 (13)	0.2500	0.03115 (9)	
O1	0.5000	0.0598 (2)	0.2500	0.0705 (10)	
O2	0.4319 (2)	0.17726 (13)	0.28901 (13)	0.0562 (6)	
O3	0.1789 (3)	0.2766 (2)	0.29302 (17)	0.1053 (11)	
O4	0.2177 (3)	0.27312 (16)	0.41595 (15)	0.0779 (8)	
O5	0.3593 (2)	0.32656 (13)	0.35615 (12)	0.0503 (5)	
O6	0.7035 (2)	0.54285 (14)	0.34535 (13)	0.0652 (7)	
O7	0.53719 (19)	0.45902 (12)	0.33599 (10)	0.0453 (5)	
O8	0.70260 (19)	0.42680 (12)	0.28267 (11)	0.0448 (5)	
OW1	0.6436 (2)	0.27739 (13)	0.37050 (11)	0.0416 (5)	
HW1A	0.7155 (19)	0.3003 (18)	0.3907 (15)	0.065 (11)*	
HW1B	0.617 (3)	0.2462 (16)	0.4032 (13)	0.063 (11)*	
N1	0.5000	0.1364 (2)	0.2500	0.0469 (9)	
N2	0.2502 (3)	0.29172 (15)	0.35287 (16)	0.0478 (6)	
N3	0.6495 (2)	0.47807 (15)	0.32205 (12)	0.0411 (6)	
N4	1.5004 (3)	0.66662 (14)	0.40465 (18)	0.0529 (7)	
H4B	1.5642	0.7007	0.4036	0.063*	
N5	1.1739 (2)	0.46981 (15)	0.46894 (14)	0.0462 (6)	
N6	1.0647 (2)	0.41729 (13)	0.46749 (12)	0.0372 (5)	
N7	0.8835 (2)	0.34651 (15)	0.44223 (14)	0.0457 (6)	
N8	0.9425 (2)	0.33038 (14)	0.51796 (14)	0.0434 (6)	
C1	1.3894 (3)	0.57624 (18)	0.47348 (16)	0.0440 (7)	
H1A	1.3802	0.5513	0.5199	0.053*	
C2	1.4894 (3)	0.6306 (2)	0.47130 (18)	0.0505 (8)	
H2A	1.5495	0.6424	0.5159	0.061*	
C3	1.4172 (3)	0.65205 (19)	0.3402 (2)	0.0524 (9)	
H3A	1.4272	0.6796	0.2951	0.063*	
C4	1.3168 (3)	0.59701 (18)	0.33902 (16)	0.0432 (7)	
H4A	1.2594	0.5858	0.2932	0.052*	
C5	1.3012 (3)	0.55803 (16)	0.40681 (16)	0.0368 (6)	
C6	1.1905 (3)	0.49910 (17)	0.40619 (17)	0.0457 (7)	
H6A	1.1356	0.4847	0.3604	0.055*	
C7	0.9585 (3)	0.39815 (18)	0.41384 (17)	0.0444 (7)	
H7A	0.9418	0.4190	0.3638	0.053*	
C8	1.0501 (3)	0.37400 (17)	0.53134 (16)	0.0417 (7)	
H8A	1.1085	0.3752	0.5780	0.050*	

### Atomic displacement parameters (Å^2^)

**Table:**

	*U*^11^	*U*^22^	*U*^33^	*U*^12^	*U*^13^	*U*^23^
Ce1	0.02878 (14)	0.03952 (14)	0.02512 (12)	0.000	0.00461 (9)	0.000
O1	0.061 (2)	0.043 (2)	0.097 (3)	0.000	−0.015 (2)	0.000
O2	0.0595 (15)	0.0588 (14)	0.0529 (13)	−0.0164 (12)	0.0170 (12)	−0.0075 (11)
O3	0.113 (3)	0.105 (2)	0.077 (2)	−0.0392 (19)	−0.0401 (19)	−0.0047 (17)
O4	0.086 (2)	0.0829 (18)	0.0775 (17)	−0.0295 (15)	0.0491 (16)	−0.0101 (14)
O5	0.0423 (13)	0.0648 (13)	0.0487 (12)	−0.0198 (11)	0.0215 (10)	−0.0070 (10)
O6	0.0732 (17)	0.0559 (14)	0.0640 (15)	−0.0263 (13)	0.0049 (13)	−0.0135 (11)
O7	0.0409 (12)	0.0547 (12)	0.0417 (11)	−0.0040 (10)	0.0107 (10)	−0.0061 (9)
O8	0.0358 (12)	0.0544 (13)	0.0450 (11)	−0.0019 (10)	0.0090 (10)	−0.0021 (10)
OW1	0.0351 (12)	0.0502 (12)	0.0361 (11)	−0.0123 (10)	−0.0035 (10)	0.0090 (10)
N1	0.036 (2)	0.052 (2)	0.047 (2)	0.000	−0.0087 (18)	0.000
N2	0.0502 (18)	0.0448 (14)	0.0493 (16)	−0.0041 (13)	0.0110 (15)	−0.0076 (12)
N3	0.0437 (15)	0.0478 (15)	0.0288 (12)	−0.0036 (13)	−0.0019 (11)	0.0036 (11)
N4	0.0437 (16)	0.0403 (15)	0.080 (2)	−0.0064 (12)	0.0264 (16)	−0.0004 (14)
N5	0.0458 (16)	0.0478 (14)	0.0457 (14)	−0.0041 (12)	0.0101 (12)	0.0040 (12)
N6	0.0311 (13)	0.0360 (12)	0.0440 (14)	−0.0087 (10)	0.0053 (11)	0.0049 (10)
N7	0.0367 (14)	0.0475 (15)	0.0499 (15)	−0.0104 (12)	−0.0008 (12)	0.0030 (11)
N8	0.0391 (14)	0.0423 (13)	0.0472 (14)	−0.0063 (12)	0.0029 (12)	0.0071 (11)
C1	0.0500 (19)	0.0494 (18)	0.0351 (15)	−0.0024 (15)	0.0143 (15)	0.0040 (13)
C2	0.046 (2)	0.0555 (19)	0.0474 (19)	−0.0051 (17)	0.0011 (16)	−0.0111 (16)
C3	0.055 (2)	0.054 (2)	0.056 (2)	0.0130 (17)	0.0306 (19)	0.0225 (16)
C4	0.0393 (18)	0.0563 (18)	0.0335 (15)	0.0104 (15)	0.0046 (14)	0.0039 (13)
C5	0.0314 (16)	0.0354 (15)	0.0449 (16)	0.0002 (13)	0.0105 (14)	−0.0005 (12)
C6	0.0474 (19)	0.0477 (18)	0.0397 (17)	0.0000 (15)	0.0011 (15)	−0.0002 (13)
C7	0.0393 (18)	0.0503 (18)	0.0412 (16)	−0.0034 (15)	0.0000 (15)	0.0071 (14)
C8	0.0376 (17)	0.0434 (17)	0.0419 (16)	−0.0050 (14)	0.0013 (14)	0.0052 (13)

### Geometric parameters (Å, °)

**Table:**

Ce1—OW1^i^	2.494 (2)	N4—C2	1.331 (4)
Ce1—OW1	2.494 (2)	N4—H4B	0.8600
Ce1—O5	2.5590 (19)	N5—C6	1.240 (3)
Ce1—O5^i^	2.5590 (19)	N5—N6	1.406 (3)
Ce1—O7	2.606 (2)	N6—C7	1.350 (3)
Ce1—O7^i^	2.606 (2)	N6—C8	1.353 (3)
Ce1—O8	2.625 (2)	N7—C7	1.296 (4)
Ce1—O8^i^	2.625 (2)	N7—N8	1.389 (3)
Ce1—O2^i^	2.634 (2)	N8—C8	1.301 (4)
Ce1—O2	2.634 (2)	C1—C2	1.360 (4)
O1—N1	1.235 (5)	C1—C5	1.384 (4)
O2—N1	1.253 (3)	C1—H1A	0.9300
O3—N2	1.199 (3)	C2—H2A	0.9300
O4—N2	1.251 (3)	C3—C4	1.362 (4)
O5—N2	1.251 (3)	C3—H3A	0.9300
O6—N3	1.221 (3)	C4—C5	1.383 (4)
O7—N3	1.264 (3)	C4—H4A	0.9300
O8—N3	1.264 (3)	C5—C6	1.485 (4)
OW1—HW1A	0.849 (10)	C6—H6A	0.9300
OW1—HW1B	0.846 (10)	C7—H7A	0.9300
N1—O2^i^	1.253 (3)	C8—H8A	0.9300
N4—C3	1.318 (4)		
			
OW1^i^—Ce1—OW1	142.88 (10)	N3—O7—Ce1	97.65 (15)
OW1^i^—Ce1—O5	106.96 (7)	N3—O8—Ce1	96.72 (15)
OW1—Ce1—O5	73.02 (8)	Ce1—OW1—HW1A	124.3 (19)
OW1^i^—Ce1—O5^i^	73.02 (8)	Ce1—OW1—HW1B	123.5 (19)
OW1—Ce1—O5^i^	106.96 (7)	HW1A—OW1—HW1B	109.8 (16)
O5—Ce1—O5^i^	179.96 (9)	O1—N1—O2	121.74 (18)
OW1^i^—Ce1—O7	139.41 (7)	O1—N1—O2^i^	121.74 (18)
OW1—Ce1—O7	76.24 (7)	O2—N1—O2^i^	116.5 (4)
O5—Ce1—O7	67.74 (6)	O3—N2—O5	122.7 (3)
O5^i^—Ce1—O7	112.29 (7)	O3—N2—O4	120.8 (3)
OW1^i^—Ce1—O7^i^	76.24 (7)	O5—N2—O4	116.5 (3)
OW1—Ce1—O7^i^	139.41 (7)	O6—N3—O7	121.6 (2)
O5—Ce1—O7^i^	112.29 (7)	O6—N3—O8	121.8 (2)
O5^i^—Ce1—O7^i^	67.74 (6)	O7—N3—O8	116.6 (2)
O7—Ce1—O7^i^	69.97 (9)	C3—N4—C2	122.5 (3)
OW1^i^—Ce1—O8	135.54 (6)	C3—N4—H4B	118.8
OW1—Ce1—O8	71.22 (7)	C2—N4—H4B	118.8
O5—Ce1—O8	111.88 (6)	C6—N5—N6	116.7 (3)
O5^i^—Ce1—O8	68.15 (7)	C7—N6—C8	105.5 (2)
O7—Ce1—O8	48.56 (6)	C7—N6—N5	134.4 (2)
O7^i^—Ce1—O8	69.72 (6)	C8—N6—N5	120.1 (2)
OW1^i^—Ce1—O8^i^	71.22 (6)	C7—N7—N8	107.4 (2)
OW1—Ce1—O8^i^	135.54 (6)	C8—N8—N7	106.8 (2)
O5—Ce1—O8^i^	68.15 (7)	C2—C1—C5	120.2 (3)
O5^i^—Ce1—O8^i^	111.88 (6)	C2—C1—H1A	119.9
O7—Ce1—O8^i^	69.72 (6)	C5—C1—H1A	119.9
O7^i^—Ce1—O8^i^	48.56 (6)	N4—C2—C1	119.3 (3)
O8—Ce1—O8^i^	104.04 (9)	N4—C2—H2A	120.4
OW1^i^—Ce1—O2^i^	68.49 (7)	C1—C2—H2A	120.4
OW1—Ce1—O2^i^	77.56 (7)	N4—C3—C4	120.5 (3)
O5—Ce1—O2^i^	113.68 (7)	N4—C3—H3A	119.7
O5^i^—Ce1—O2^i^	66.29 (7)	C4—C3—H3A	119.7
O7—Ce1—O2^i^	151.86 (7)	C3—C4—C5	119.2 (3)
O7^i^—Ce1—O2^i^	128.09 (7)	C3—C4—H4A	120.4
O8—Ce1—O2^i^	112.40 (7)	C5—C4—H4A	120.4
O8^i^—Ce1—O2^i^	138.12 (7)	C4—C5—C1	118.4 (3)
OW1^i^—Ce1—O2	77.56 (7)	C4—C5—C6	119.4 (3)
OW1—Ce1—O2	68.49 (7)	C1—C5—C6	122.2 (2)
O5—Ce1—O2	66.29 (7)	N5—C6—C5	117.6 (3)
O5^i^—Ce1—O2	113.68 (7)	N5—C6—H6A	121.2
O7—Ce1—O2	128.09 (7)	C5—C6—H6A	121.2
O7^i^—Ce1—O2	151.86 (7)	N7—C7—N6	110.1 (2)
O8—Ce1—O2	138.12 (7)	N7—C7—H7A	124.9
O8^i^—Ce1—O2	112.40 (7)	N6—C7—H7A	124.9
O2^i^—Ce1—O2	47.72 (10)	N8—C8—N6	110.1 (3)
N1—O2—Ce1	97.88 (19)	N8—C8—H8A	124.9
N2—O5—Ce1	126.02 (17)	N6—C8—H8A	124.9
			
OW1^i^—Ce1—O2—N1	72.13 (12)	O7^i^—Ce1—O8—N3	76.92 (14)
OW1—Ce1—O2—N1	−92.60 (13)	O8^i^—Ce1—O8—N3	41.97 (12)
O5—Ce1—O2—N1	−172.83 (15)	O2^i^—Ce1—O8—N3	−159.10 (14)
O5^i^—Ce1—O2—N1	7.16 (15)	O2—Ce1—O8—N3	−108.18 (15)
O7—Ce1—O2—N1	−143.28 (10)	Ce1—O2—N1—O1	180.0
O7^i^—Ce1—O2—N1	93.88 (17)	Ce1—O2—N1—O2^i^	0.0
O8—Ce1—O2—N1	−75.96 (15)	Ce1—O5—N2—O3	−24.5 (4)
O8^i^—Ce1—O2—N1	135.54 (11)	Ce1—O5—N2—O4	155.8 (2)
O2^i^—Ce1—O2—N1	0.0	Ce1—O7—N3—O6	173.1 (2)
OW1^i^—Ce1—O5—N2	14.3 (2)	Ce1—O7—N3—O8	−6.7 (2)
OW1—Ce1—O5—N2	−126.8 (2)	Ce1—O8—N3—O6	−173.2 (2)
O5^i^—Ce1—O5—N2	−56 (100)	Ce1—O8—N3—O7	6.6 (2)
O7—Ce1—O5—N2	151.4 (2)	C6—N5—N6—C7	−10.8 (4)
O7^i^—Ce1—O5—N2	96.0 (2)	C6—N5—N6—C8	170.9 (3)
O8—Ce1—O5—N2	172.2 (2)	C7—N7—N8—C8	0.5 (3)
O8^i^—Ce1—O5—N2	75.3 (2)	C3—N4—C2—C1	0.3 (5)
O2^i^—Ce1—O5—N2	−59.2 (2)	C5—C1—C2—N4	0.8 (5)
O2—Ce1—O5—N2	−53.4 (2)	C2—N4—C3—C4	−1.6 (5)
OW1^i^—Ce1—O7—N3	−111.82 (16)	N4—C3—C4—C5	1.6 (4)
OW1—Ce1—O7—N3	80.77 (15)	C3—C4—C5—C1	−0.4 (4)
O5—Ce1—O7—N3	157.79 (16)	C3—C4—C5—C6	178.9 (3)
O5^i^—Ce1—O7—N3	−22.19 (16)	C2—C1—C5—C4	−0.8 (4)
O7^i^—Ce1—O7—N3	−76.37 (14)	C2—C1—C5—C6	179.9 (3)
O8—Ce1—O7—N3	3.83 (13)	N6—N5—C6—C5	177.0 (2)
O8^i^—Ce1—O7—N3	−128.32 (16)	C4—C5—C6—N5	−175.0 (3)
O2^i^—Ce1—O7—N3	58.9 (2)	C1—C5—C6—N5	4.4 (4)
O2—Ce1—O7—N3	128.58 (14)	N8—N7—C7—N6	−0.2 (3)
OW1^i^—Ce1—O8—N3	119.31 (14)	C8—N6—C7—N7	−0.1 (3)
OW1—Ce1—O8—N3	−91.83 (15)	N5—N6—C7—N7	−178.6 (3)
O5—Ce1—O8—N3	−29.79 (16)	N7—N8—C8—N6	−0.6 (3)
O5^i^—Ce1—O8—N3	150.24 (15)	C7—N6—C8—N8	0.5 (3)
O7—Ce1—O8—N3	−3.82 (13)	N5—N6—C8—N8	179.2 (2)

Symmetry codes: (i) −*x*+1, *y*, −*z*+1/2.

### Hydrogen-bond geometry (Å, °)

**Table:**

*D*—H···*A*	*D*—H	H···*A*	*D*···*A*	*D*—H···*A*
OW1—HW1A···N7	0.85 (2)	1.96 (2)	2.805 (3)	176 (3)
OW1—HW1B···N8^ii^	0.85 (2)	2.03 (3)	2.876 (3)	178 (3)
N4—H4B···O4^iii^	0.86	1.95	2.804 (3)	172
N4—H4B···N2^iii^	0.86	2.69	3.518 (4)	162

Symmetry codes: (ii) −*x*+3/2, −*y*+1/2, −*z*+1; (iii) *x*+3/2, *y*+1/2, *z*.
